# Extremely preterm neonates have more *Lactobacillus* in meconium than very preterm neonates – the *in utero* microbial colonization hypothesis

**DOI:** 10.1080/19490976.2020.1785804

**Published:** 2020-07-13

**Authors:** Juliana Morais, Cláudia Marques, Diana Teixeira, Catarina Durão, Ana Faria, Sara Brito, Manuela Cardoso, Israel Macedo, Esmeralda Pereira, Teresa Tomé, Conceição Calhau

**Affiliations:** a Nutrition and Metabolism, Faculdade de Ciências Médicas|NOVA Medical Schoo|, Universidade NOVA de Lisboa, Lisboa, Portugal; b CINTESIS, Center for Health Technology Services Research, Porto, Portugal; c Comprehensive Health Research Centre, Universidade NOVA de Lisboa, Lisboa, Portugal; d Unidade Universitária Lifestyle Medicine José de Mello Saúde by NOVA Medical School, Lisboa, Portugal; e EPIUnit - Institute of Public Health, Universidade do Porto, Porto, Portugal; f Neonatal Intensive Care Unit, Liutrition and Dietetics Unit, Centro Hospitalar Universitário de Lisboa Central, Maternidade Dr. Alfredo da Costa, Cesboa, Portugal; g Nutrition and Dietetics Unit, Maternidade Dr. Alfredo da Costa, Centro Hospitalar Universitário de Lisboa Central, Lisboa, Portugal; h Neonatology Department, Maternidade Dr. Alfredo da Costa, Centro Hospitalar Universitário de Lisboa Central, Lisboa, Portugal

**Keywords:** *Lactobacillus*, meconium, maternal microbiota, preterm neonates

## Abstract

Growing evidence suggests that maternal microbiota can influence the neonates’ gut colonization. However, the mechanisms of vertical bacterial transmission remain poorly defined. We believed that the first colonizers of the newborn come from the mother’s gut and vagina during pregnancy and that this is independent of the mode of delivery. We conducted an observational longitudinal study to evaluate the link between the maternal gut microbiota and the meconium’s microbiota in extremely and very preterm neonates. Bacterial DNA was extracted from samples and specific bacterial groups were quantified by RT-PCR. In this cohort of 117 preterm neonates, we detected bacterial DNA in 88% of meconium samples. Meconium microbiota of neonates born after 28 gestational weeks (very preterm neonates) showed stronger correlations with their mothers’ fecal microbiota. However, neonates born before 28 gestational weeks (extremely preterm neonates) had more *Lactobacillus* – genus that dominated the vaginal microbiota – than very preterm neonates, regardless of the mode of delivery. Collectively, these data support the hypothesis that maternal bacteria from the gut and vagina can play a role in shaping neonates’ gut microbiota and that mother-to-infant bacterial transmission is a controlled and time-specific process. ClinicalTrials.gov Identifier: NCT03663556

## Introduction

Although the colonization process is a dogma between the “sterile womb” and “*in utero* colonization” hypotheses,^1^ mother-to-infant microbiota transmission may be one of the mechanisms linking the intrauterine environment and the susceptibility to disease in later (and even in early) life. The intrauterine colonization hypothesis became stronger when animal studies conducted by Jiménez *et al*^2^ demonstrated the maternal-fetal transfer of microbes.^2^ Using labeled *Enterococcus faecium* isolated from the breast milk of healthy woman, pregnant mice were orally inoculated and the pups were then delivered by C-section.^2^ The labeled strain was detected in the amniotic fluid^2^ of these animals and in the pups’ meconium.^3^ Later, two independent studies conducted by Aagaard et al.^4^ and Stout et al.^5^ detected bacterial content in placenta samples (n = 320 and n = 195, respectively) of women who gave birth prematurely and also in women who had term healthy pregnancies.^4,5^ A recent work “confidently” detected bacterial DNA in placentas of 13 from 16 spontaneous preterm births and in 18 of 22 term unlabored cesareans, with no significant differences between preterm and term deliveries.^6^ However, the evidence is unclear. Lauder *et al*.^7^ did not find any difference between placental samples and negative controls.^7^ Nevertheless, even if the “sterile womb” hypothesis is correct and the *in utero* colonization occurs only under certain circumstances (subclinical conditions), it is still important to understand how it happens in order to optimize mother, fetus and infant health outcomes.

Despite some contradictory works, it is assumed that the mode of delivery may play a decisive role in the development and growth of the newborns, since the bacteria present in the fetal gastrointestinal tract can influence the development of the immune system and therefore have relevant health consequences.^8^ While neonates born by vaginal delivery receive a microbiota similar to that of the maternal vagina (through the passage in cervix and vagina), C-section delivered neonates are enriched in skin microbiota, hospital staff and environment microorganims.^9^ Recently, it was reported that the mode of delivery had a temporary and small effect on neonates’ gut microbiota and the gestational age seems to be the main driver.^10^ The microbiota composition of preterm infants is significantly different from that of the full-term infants.^11,12^ Preterm infants – defined as an infant born before 37 weeks of pregnancy, which includes very preterm infant born between 28 and 32 weeks and extremely preterm born before 28 weeks^13^ – present an immature intestinal microbiota with marked vulnerability to dysbiosis, altered microbial abundance and diversity, as well as progressive acquisition of bacteria.^14^

A recent study with term infants concluded that vertical microbiota transmission is a physiological process and even though these infants present many microbial strains that maternal microbiota cannot explain, there is strong evidence supporting the microbial transmission from multiple maternal sources to infants.^15^ However, the evidence on vertical microbiota transmission in prematurely delivered infants is very scarce.

In this regard, we conducted an observational longitudinal study to evaluate the link between the maternal gut microbiota and the extremely and very preterm neonates’ meconium microbiota. By comparing extremely preterm neonates with very preterm neonates, it was possible to study the effects of gestational age on the colonization of the meconium.

## Results

**Figure 1.**
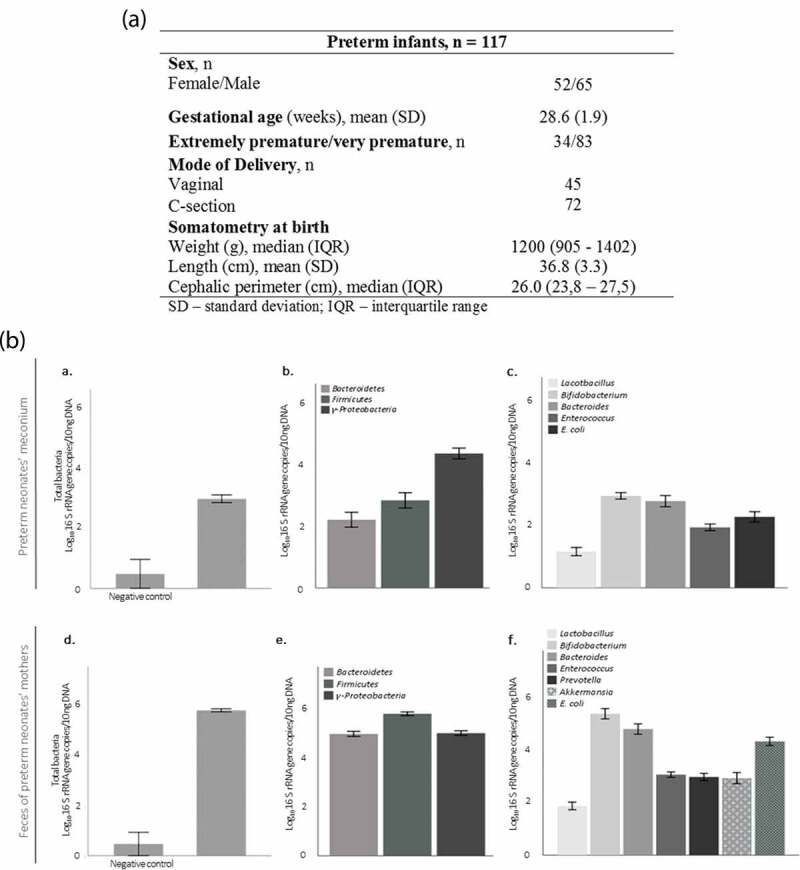
Sample characterization. (a) Clinical characterization of preterm neonates. (b) Specific bacterial group levels in neonates’ meconium (a, b, c), and in their mothers fecal samples (d, f, e). Negative controls for sample collection, DNA extraction and RT-PCR (n = 5). Data are expressed as mean ± SEM.

From a total of 159 preterm neonates recruited consecutively in the NICU between May 2017 and April 2019, 117 preterm neonates (< 32 weeks gestational age) were included in this study. Their respective mothers (n = 93) were also enrolled in this study. Sociodemographic and clinical data of preterm neonates is reported in Figure 1a.

### Characterization of meconium microbiota of preterm neonates

In this cohort, 88% of the meconium samples collected before 72 post-natal hours were colonized (range: 0.790–5.441 log_10_ 16 S rRNA gene copies/10 ng of DNA) (Figure 1b.a). *Proteobacteria* was the most abundant phylum in the meconium of preterm neonates (Figure 1b.b). Of all genera analyzed in meconium samples, *Bifidobacterium* was the most abundant genus (Figure 1b.c). From mothers, 64 postpartum fecal samples were analyzed (Figure 1b.d, b.e,b.f). Similar to preterm neonates, *Bifidobacterium* was the most abundant genus in the mothers’ samples. The content of all the analyzed bacterial groups was higher in the mothers’ samples than in meconium (*p* < .05) with the exception of *Lactobacillus* (*p* = .146) (Figure 1b.c,b.f).

### Mother-to-infant bacterial transmission in preterm neonates

**Figure 2.**
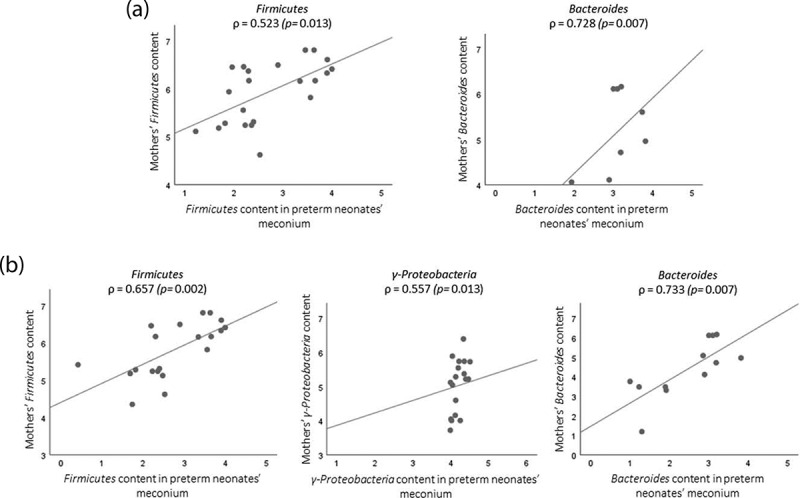
Scatterplots showing the association between mothers’ microbiota and their neonates’ microbiota (Spearman's correlation): (a) represent correlation between very preterm neonates and their mothers; (b) correlations of mother-infants pairs delivered by C-section.

In order to determine the effect of gestational age on bacterial transfer from mother to offspring, Spearman’s correlation analysis was performed between preterm neonates’ meconium and their mothers’ microbiota. Interestingly, no correlations were found between the microbes analyzed in the mothers’ fecal samples or the microbes analyzed in the extremely preterm neonates’ meconium (data not shown). However, in preterm neonates born during the third trimester (between 28 and 32 weeks gestation), the content of *Firmicutes* and *Bacteroides* correlated positively and strongly [ρ = 0.523 (*p* = .013) and ρ = 0.728 (*p* = .007), respectively (n = 24)] with the mothers’ *Firmicutes* and *Bacteroides* content, suggesting that mother-to-infant gut bacterial transmission may increase markedly after the 28^th^ week of gestation (Figure 2a).

Regarding the mode of delivery, among 14 vaginal deliveries, no correlation was observed between mothers’ and neonates’ microbiota. These results were quite unexpected since colonization of the preterm neonates with maternal gut microbiota was likely to occur during vaginal delivery due to the proximity of the rectum. On the other hand, among 21 C-section deliveries, mothers’ microbiota and preterm neonates’ meconium were positively correlated: *Firmicutes, γ-Proteobacteria* and *Bacteroides* [ρ = 0.657 (*p* = .002), ρ = 0.557 (*p* = .013) and ρ = 0.733 (*p* = .007), respectively].

### The gestational age and microbiota acquisition

**Figure 3.**
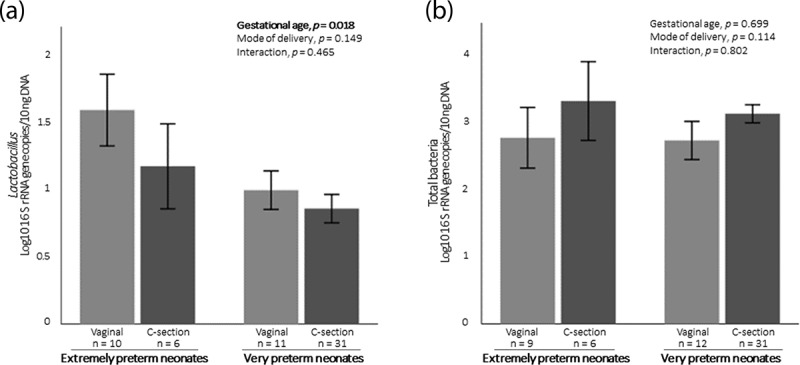
(a) Content of *Lactobacillus* in preterm neonates’ meconium regarding gestational age and mode of deliver. (b) Content of total bacteria in preterm neonates’ meconium regarding gestational age and mode of delivery. Data are expressed as mean ± SEM.

Since mother-to-infant microbiota transmission was dependent on gestational age and mode of delivery, we decided to compare the meconium microbiota of extremely preterm neonates with that of very preterm neonates, in accordance with their mode of delivery. The analyzed bacterial groups in meconium did not differ between extremely preterm and very preterm neonates, except from *Lactobacillus*. Extremely premature newborns’ meconium had higher amounts of *Lactobacillus* than that of very preterm neonates (1.442 ± 0.822 vs. 0.899 ± 0.561, *p* = .018). Remarkably, it was observed that extremely preterm neonates had more *Lactobacillus* in their meconium regardless of whether they were born by vaginal delivery or C-section (Figure 3a). This pattern was exclusive to *Lactobacillus* as it was not observed for total bacteria content (Figure 3b) or any other analyzed bacterial group (data not shown).

## Discussion

Understanding the role of the intrauterine environment on fetal microbiota and the impact of very early postnatal factors on neonates’ microbiota is essential for bacterial modulation through clinical interventions such as maternal diet, exposure to antibiotic, probiotics and prebiotics, or even fecal transplantation. Since meconium is a biological material formed during the gestation, it has been considered a very useful source of information that reflects the *in utero* microbial environment.^16^

The findings presented in this brief report indicate that almost all meconium samples were colonized; thus it can be assumed that neonates’ meconium may have bacterial DNA prior to birth. It has been reported that mothers can be responsible for the transference of (some) these microbes to the fetus, as well as their metabolites and other molecules that shape the offspring’s innate immune system.^17^ Two mechanisms have been proposed to explain the mother-to-infant bacterial transmission: the hematogenous bacterial route of bacteria from the gastrointestinal tract (oral cavity and gut); and the ascension of bacteria from vaginal microbiota. Both routes argued that bacteria enter into the blood circulation and are incorporated into the placenta decidua and, consequently, into the developing fetus via cord blood and amniotic fluid.^9^

Our results showed that very preterm neonates (born with more than 28 gestational weeks) presented stronger bacterial correlations with their mothers’ gut microbiota than extremely preterm neonates. The stronger correlations found between *Firmicutes* from mothers’ feces and meconium of very preterm neonates are consistent with lower abundance *Firmicutes* in low birth weight neonates’ placenta.^18^ Taking into account that it is during the last trimester of pregnancy (> 28 weeks) that the fetus swallows large quantities of amniotic fluid,^19^ and that bacterial DNA in amniotic fluid has previously been detected,^20^ it is tempting to postulate that there are higher amounts of maternal bacterial content in the meconium of neonates born in this gestational period. Based on this, we suggested that bacterial translocation via the hematogenous route is a process that may increase over gestation.

Contrary to what was observed between mothers and their infants born by vaginal delivery, bacterial correlations were stronger for preterm neonates delivered by C-section. Passage through the vaginal canal promoted a distinct colonization that may have mitigated the effect of vertical microbial transmission during pregnancy. The vaginal microbiota is known to be different from the gut microbiota and is dominated by *Lactobacillus* species^21^ that are essential to produce lactic acid and, consequently, to maintain a low vaginal pH preventing dysbiosis and infection that could reach the fetus.^22^ The vaginal microbiota is dynamic and changes based on gestational age.^23^ Vaginal introitus and midvaginal samples collected between the 24^th^ and 28^th^ gestational weeks showed greater richness and diversity than samples collected between the 28^th^ and 32^nd^ weeks.^23^ Therefore, according to the theory of vaginal bacteria ascension, it is possible to speculate that more *Lactobacillus* may reach the fetus in the 24^th^-28^th^ than in the 28^th^-32^nd^ weeks of gestation. In line with this, we observed that extremely preterm neonates (born before 28 weeks) had more *Lactobacillus* in their meconium compared to very preterm neonates (born between 28 and 32 gestational weeks). In addition, we observed that this difference was not influenced by the mode of delivery. Although the literature shows preterm neonates born by a vaginal delivery tended to have higher levels of *Lactobacillus*,^24^ our results showed that even when born by C-section, extremely preterm neonates had higher levels of *Lactobacillus* than very preterm neonates. Ardissone *et al* also found that preterm neonates’ meconium had greater relative abundance of *Lactobacillus* compared to term neonates and that the mode of delivery did have a minor effect.^25^ The lower reproductive tract is well characterized as being predominantly colonized by *Lactobacillus* as well as endometrium, which despite having a much more diverse microbiota, is also dominated by *Lactobacillus*.^26^ The microbial presence in the uterine decidua may support the vaginal ascending route leading to *in utero* colonization through bacterial transmission during placentation. Indeed, the presence of bacteria in the placenta and amniotic liquid is consistent with the fact that the neonate microbiota at birth was homogeneously distributed across the body (skin, nares, oral cavity and gut) regardless of the mode of delivery.^27^

**Figure 4.**
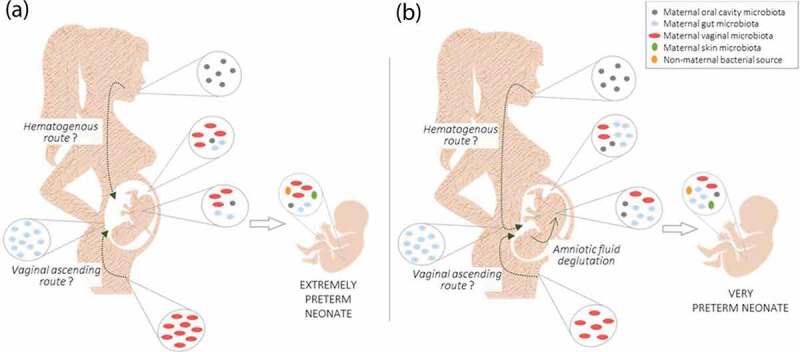
Controlled and time-specific mother-to-infant bacterial transmission: (a), the vertical bacterial transmission starts mainly with *Lactobacillus* (predominantly from vaginal microbiota) via the vaginal ascending route; (b), during the third trimester, maternal gut bacteria is predominantly transmitted to the fetus intestine via the hematogenous route and through deglutination of amniotic fluid.

Taken together, these findings lead us to support the theory of vertical bacterial transmission that is represented in Figure 4: mother-to-infant bacterial transmission occur simultaneously through the two routes mentioned above, but differently in the course of pregnancy. Initially, during the 24^th^ to 28^th^ weeks of gestation, vaginal microbiota (dominated by *Lactobacillus*) is mainly transmitted to placental/amniotic fluid via vaginal ascending (Figure 4a); and later, during the third trimester, maternal gut bacteria is predominantly transmitted to fetus intestine – a process that occurs as the maternal gut permeability is higher at this time,^28^ but also because it is during the last trimester that the fetus swallows large quantities of amniotic fluid^19^ (Figure 4b).

Studies to evaluate the microbiota in extremely and very preterm neonates are very scarce since this is a particularly vulnerable study population; it is particularly challenging to work with as the meconium sample collection within 72 hours of life avoids eventual bacterial colonization due to external factors (NICU environment and medical and nursing contact). The use of appropriate negative controls and the bacterial DNA analysis by real time-PCR allowed an absolute quantification of the bacteria of interest in these infants giving strength to these results. The main limitation of this study is the absence of maternal vaginal samples for microbial analysis. However, the maternal fecal samples lead us to extrapolate interesting conclusions.

Altogether these findings open new possibilities in future research to consider the role played by bacteria from the maternal gut and vagina in shaping newborns’ gut microbiota with different gestational age.

## Material and methods

This study was approved by the Ethics Committee of Centro Hospitalar Universitário de Lisboa Central (Ref. 443/2017) and by the Ethics Committee of NOVA Medical School|Faculdade de Ciências Médicas, Universidade NOVA de Lisboa (NMS|FCM). The study was conducted in accordance with the ethical principles expressed in the Declaration of Helsinki, the Portuguese law and Good Clinical Practice guidelines.

### Study design

The FEEDMI study was an observational longitudinal study conducted at the NICU of Maternidade Dr. Alfredo da Costa (MAC) and NMS|FCM. The study is registered on the *ClinicalTrials.gov* platform, with the registration number NCT03663556. The detailed study protocol is published elsewhere.^29^

### Participant recruitment

Very preterm neonates (< 32 weeks gestational age) hospitalized in the NICU of MAC were recruited within the first 24 hours after birth. Written informed consents were obtained for each preterm infant after explaining the entire study protocol to their legal representatives.

### Sample collection and analysis

Meconium, the newborn’s first intestinal discharge, was collected directly from the infant’s diapers and placed in sterile tubes within the first 72 hours after birth by the nursing team of MAC’s Neonatology Uni. Mothers were also asked to collect their own fecal samples with the appropriate stool collection kit provided (EasySampler®). Bacterial DNA was extracted and purified from stool samples using NZY Tissue gDNA Isolation Kit (nzytech, Lisbon, Portugal). Specific bacterial populations were analyzed by quantitative real-time PCR using the LightCycler instrument (Roche Applied Science, Indianapolis, ID, USA). Although whole-genome or 16 S rRNA sequencing have become the methods of choice for microbiome analysis, the use of real-time PCR allowed a rapid and absolute quantification of the specific bacterial populations of interest in these neonates. The relative quantification of the taxa provided by sequencing can compromise the interpretation of microbiota alterations, because if a single taxon changes in relative abundance, the relative abundances of other taxa will also change. In addition, in the particular case of *Lactobacillus* which is expected to be present in the gut at very low counts, results from sequencing could underestimate the absolute amount of this genus in the analyzed samples.^30^ Microbiota results are expressed as log_10_ 16 S rRNA gene copies/10 ng of DNA.

These analyzes were conducted using all the appropriate negative controls. Negative control for sample collection was performed as follows: an empty tube (the same tube used for collecting meconium and feces samples) was opened inside the neonate’s incubator and the spatula was passed through the diaper; the tube was stored under the same conditions as the others; in the lab, 200 μL of ultrapure water was added to the tube; and DNA was extracted. DNA amplification of negative controls was performed in duplicate and samples with lower levels than the negative were not included in the data analysis. Results of negative controls are shown in Figure 1b (n = 5).

### Statistical analysis

Statistical analysis was performed by SPSS software, version 25 (IBM SPSS Statistics corporation, Chicago, IL, USA). Comparisons were made between groups using Mann-Whitney test. Spearman’s correlation test was used to examine the relationship between the meconium’s microbiota and mothers’ microbiota. Two-way ANOVA was used to determine the main effects of gestational age (extremely preterm vs very preterm), mode of delivery (vaginal vs C-section) and their interaction on *Lactobacillus* and total bacteria content in meconium. Data are expressed as mean ± standard error of the mean (SEM). The differences were considered statistically significant when *p* < .05.
